# A case of an intraabdominal, but extrahepatic ruptured percutaneous transhepatic biliary drainage and its following rescue. A case report and literature review

**DOI:** 10.1016/j.radcr.2024.08.035

**Published:** 2024-09-03

**Authors:** Mohammed Misbahuddin-Leis, Muzaffer Ankolvi, Krisztina Dubasz, Manisha Mishra, Thomas Mueller, Oleg Vorontsov, Christian Graeb, Boris Radeleff

**Affiliations:** a Medical Faculty Heidelberg, Heidelberg University, Heidelberg, Baden Wuerttemberg, Germany; b Department of Diagnostic and Interventional Radiology, Sana Klinikum Hof GmbH, Hof, Germany; c Department of Visceral and Abdominal Surgery, Sana Klinikum Hof GmbH, Hof, Germany; d Department of Gastroenterology, Hepatology, Infectiology, Hematology and Oncology, Sana Klinikum Hof GmbH, Hof, Germany

**Keywords:** IR, Percutaneous transhepatic biliary drainage, Biliary drains, Biliary interventions, Complications, Infection

## Abstract

Percutaneous transhepatic biliary drainage is a well-established technique for the treatment of biliary obstruction in patients with failed endoscopic approaches. We report on an 82-year-old man with a history of cholangiocarcinoma treated with pancreaticoduodenectomy who presented with recurrent cholangitis and sepsis. Percutaneous transhepatic biliary drainage was performed after unsuccessful endoscopic retrograde cholangiography, which initially improved his condition. However, due to an accidental dislodgement, there was an intra-abdominal fracture of the drain which led to biliary peritonitis and clinical deterioration. The fractured intrahepatic drain was successfully extracted in our angio suite, and a novel subcutaneous fixation technique was introduced to prevent similar occurrences in the future. This case study signifies the role of interventional radiology in the management of percutaneous transhepatic biliary drainage complications and the importance of preventative measures to avoid dislodgement.

## Introduction

Percutaneous transhepatic biliary drainage (PTBD) is a routinely used intervention introduced almost nearly 4 decades ago and was first reported in 1974 [1] in patients in whom endoscopic retrograde cholangiography (ERCP) was unsuccessful or not possible [2]. It is primarily used in the treatment of benign, malignant and postoperative stenoses, of inflammatory-related cholestasis and also used to treat postoperative and post-transplantation bile leaks of e.g. the biliodigestive anastomosis [3]. PTBD placement is a minimally invasive procedure performed in an angio-suite usually after local anaesthesia and i.v. analgosedation under combined sonographic and fluoroscopic guidance, in which the PTBD is placed via the biliary system into the small intestine. PTBD placement can cause relevant complications (3%-10%) [4,5] with the most common as cholangitis, hemorrhage, pneumothorax, pseudoaneurysm, fistula, and bile leak to name the most common reported complications [6].

Among these complications, rupture of the biliary drainage is not so rare (1%-5%) [7] but occurs normally extracorporal due to direct damage to the material of the drainage (e.g. during fixation with the suture needle or during nursing care on the ward). In contrary intrabdominal rupture of the drainage is a very rare complication scarcely reported in the literature und occurs in most cases intrahepatic (0.3%) [7]. We henceforth report our experience with an elderly patient with recurrent pyogenic cholangitis (RPC), who had a PTBD placed for sepsis. The patient accidentally pulled on the PTBD, resulting in a complete intraabdominal but extrahepatic rupture of the drain, which worsened his clinical situation.

## Case report

**Fig. 1:**
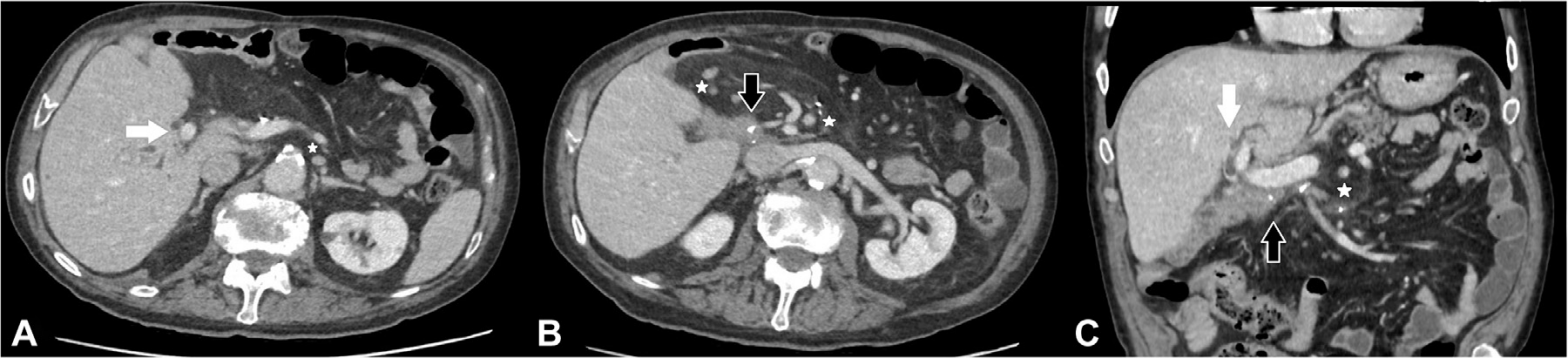
(A-C) Patient with a history of cholangiocarcinoma treated by pylorus-preserving pancreaticoduodenectomy with biliodigestive anastomosis. After several recurrent episodes of cholangitis, CT shows intrahepatic cholestasis with cholangitis of the distal courses of the right and left hepatic ducts (white arrows). Inflammatory changes of the BDA without evidence of insufficiency (black arrows). There is also an increased number of paraaortic, interaortocaval and retrocaval lymph nodes measuring almost 12 mm (white star).

An 82-year-old male with known bile duct cancer (TNM classification: pT3 L0 V0 Pn1 pN0 (0/17) R0 G3) treated by pylorus-preserving pancreaticoduodenectomy with biliodigestive anastomosis (BDA) creation 4 years ago in 2016, presented to the emergency department after multiple recurrent episodes of cholangitis over a 5-months period. At the time of presentation, he was clinically septic with a temperature of over 39 degrees Celsius and a Glasgow Coma Scale of 12 without abdominal pain or discomfort. Laboratory parameters on day 1 showed elevated CRP of 10 mg/L (> 5 mg/L), WBC of 10.9 G/L (3.7-9.9 G/L), and liver parameters (serum bilirubin: 4.3 mg/dL (>1.2 mg/dL); GPT: 160 U/L (>50 U/L); ALP: 971 U/L (40-130 U/L). A multiphase i.v. contrast enhanced MDCT (Revolution HR, GE Healthcare, USA with 100 mL i.v. contrast medium) of the abdomen (Fig. 1) at the next day revealed intrahepatic cholestasis with enhancement of the bile duct wall distal to the BDA and stranding of the fat tissue around the BDA as a result of ongoing cholangiosepsis.

Because of futile attempts by ERCP due to the altered anatomy (status post pylorus-preserving pancreaticoduodenectomy) in an interdisciplinary discussion (surgery, gastroenterology, and radiology), an interventional radiological approach using PTBD was decided for the next day.

**Fig. 2:**
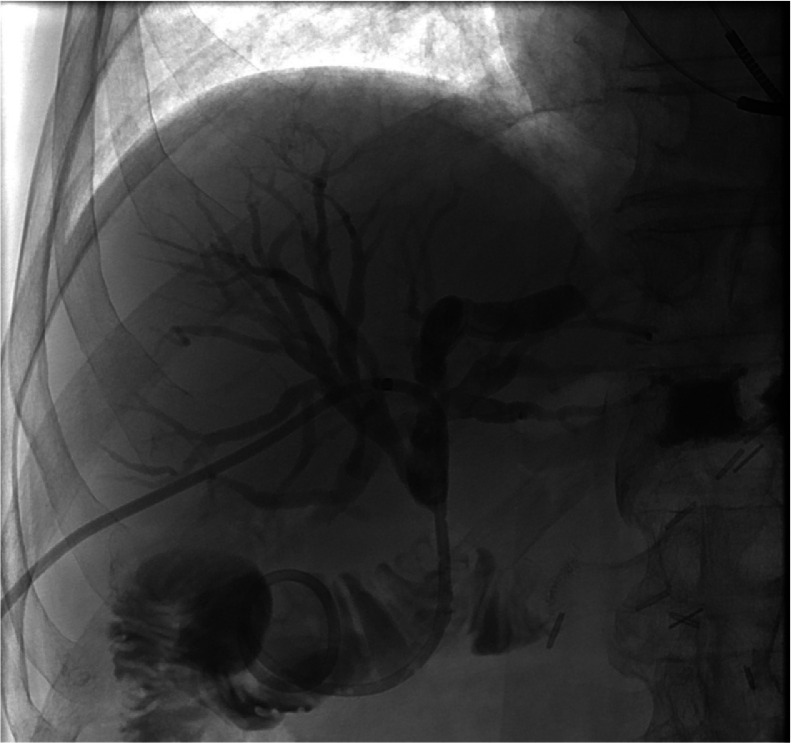
Due to intrahepatic cholestasis, ongoing cholangiosepsis, and futile attempts at ERCP caused by altered anatomy, a fluoroscopically guided PTBD from the right side using a Neff set (Cook, USA) was planned. After a successful puncture and injection of contrast medium into the bile duct system, intrahepatic cholestasis was depicted. An 8F pigtail-configured bile duct drain (Cook, USA) was then inserted via a 0.035″ stiff heavy-duty interchangeable wire (Amplatzer Super Stiff, BSCI, USA), followed by its configuration and subcutaneous fixation.

The following morning (day 3), a combined ultrasound/fluoroscopy-guided PTBD was placed into the right-sided biliary system in the angio-suite according to the guidelines in Angiofibel [8]: after application of local anaesthesia (10 ml Prilocainhydrochlorid, Xylonest® 1 %, Aspen Germany GmbH), the right-sided bile duct was punctured using a Neff set (Neff Percutaneous Access Set™, Cook, USA) under ultrasound guidance (Serie Acuson Freestyle, Siemens, USA). Subsequent contrast administration showed high-grade stenosis in the proximal common bile duct (CBD), extending directly to the bifurcation. We placed an 8F PTBD (PFM Medical AG, Germany) as an internal-external drainage (see Fig. 2). The patient started to improve immediately clinically after the procedure (laboratory results on day 4: CRP: 58 mg/L; serum bilirubin: 9.4 mg/dL; GPT: 147 U/L; ALP: 616 U/L).

**Fig. 3:**
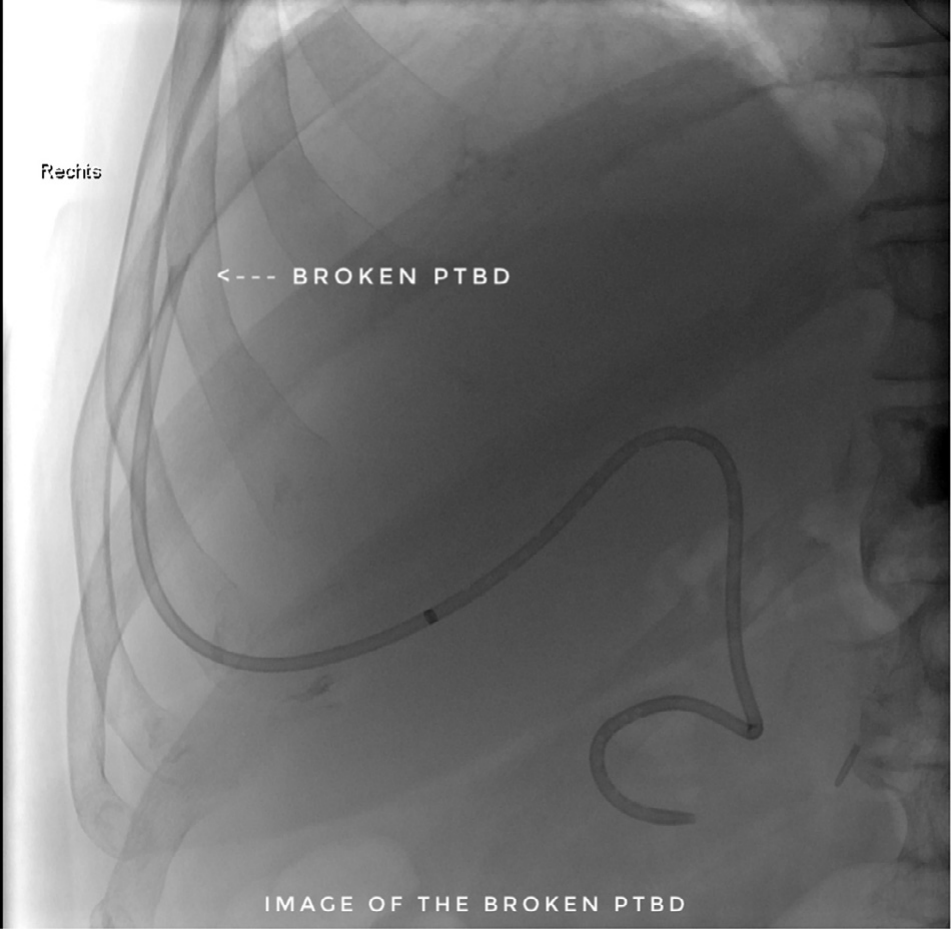
After the initial PTBD placement (fifth day), the patient accidentally pulled the PTBD, resulting in its complete rupture. A fluoroscopy image shows a fractured PTBD, with its proximal end located extrahepatically in the intraabdominal region (white arrowhead).

The following day on the ward (day 5), the patient accidentally pulled on the 8F PTBD resulting in a complete rupture of the drain outside of the body with its proximal end lying extrahepatically in the intraabdominal portion (see Fig. 3) leaking into the peritoneum. As a result of the bile leak caused by the rupture of the PTBD, he developed biliary peritonitis and was immediately transferred to our intensive care unit for further management.

**Fig. 4:**
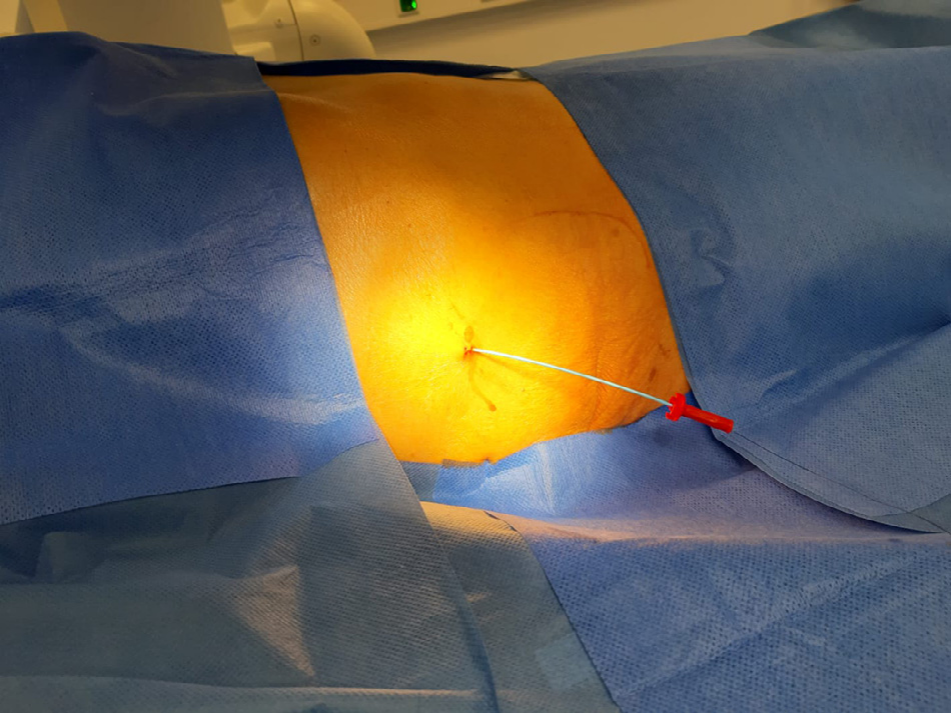
The fractured thread of the PTBD, still intraabdominally located, was passed through the dilator of a 4F sheath (Terumo Corp., Japan).

**Fig. 5:**
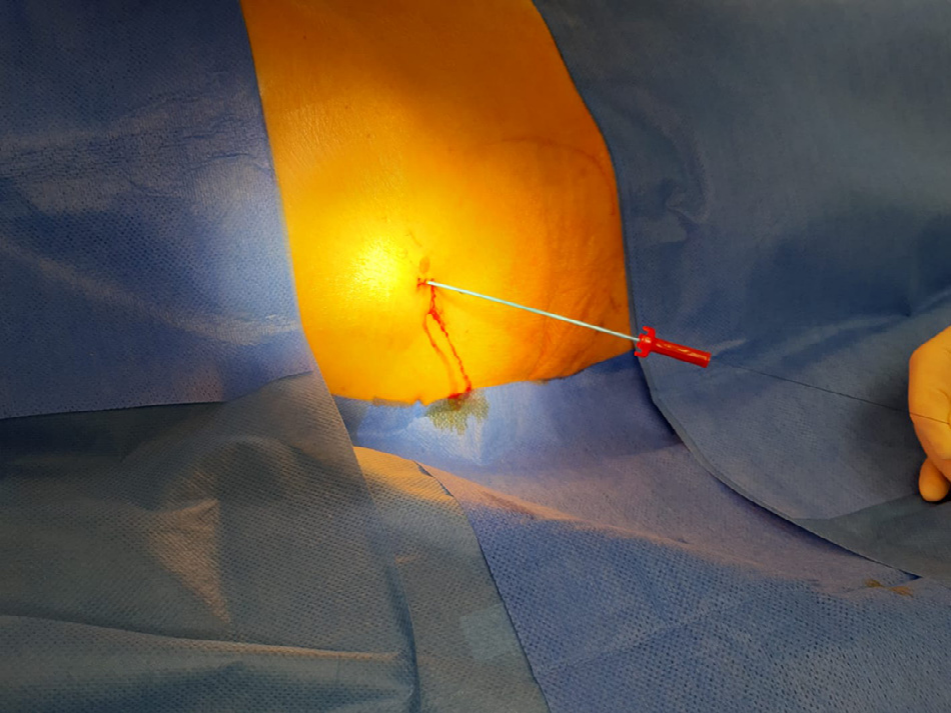
The dilator was then introduced over the tightened end of the thread to entrap the intraabdominal portion of the fPTBD.

**Fig. 6:**
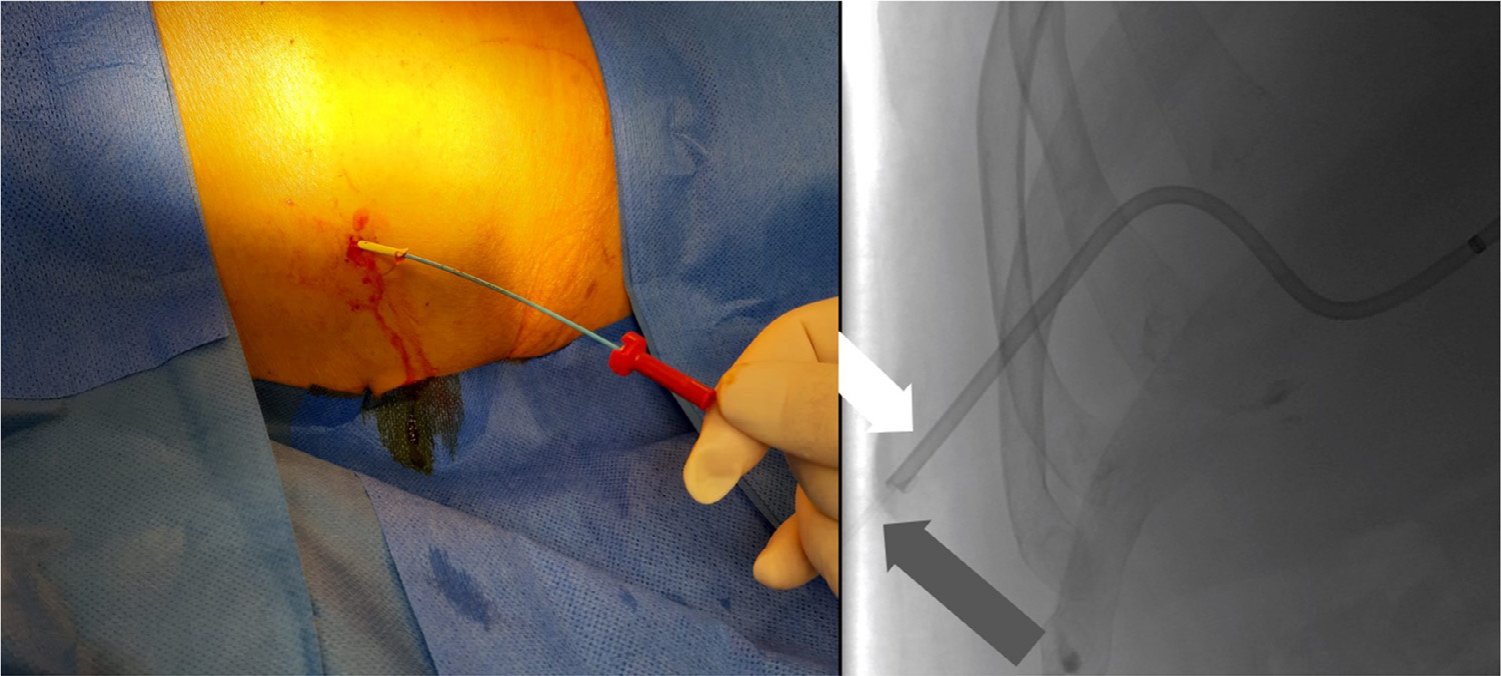
The dilator was firmly pushed into the intraabdominal end of the fPTBD. Fluoroscopic image showing the inserted dilator into the fPTBD (white and black arrows).

**Fig. 7:**
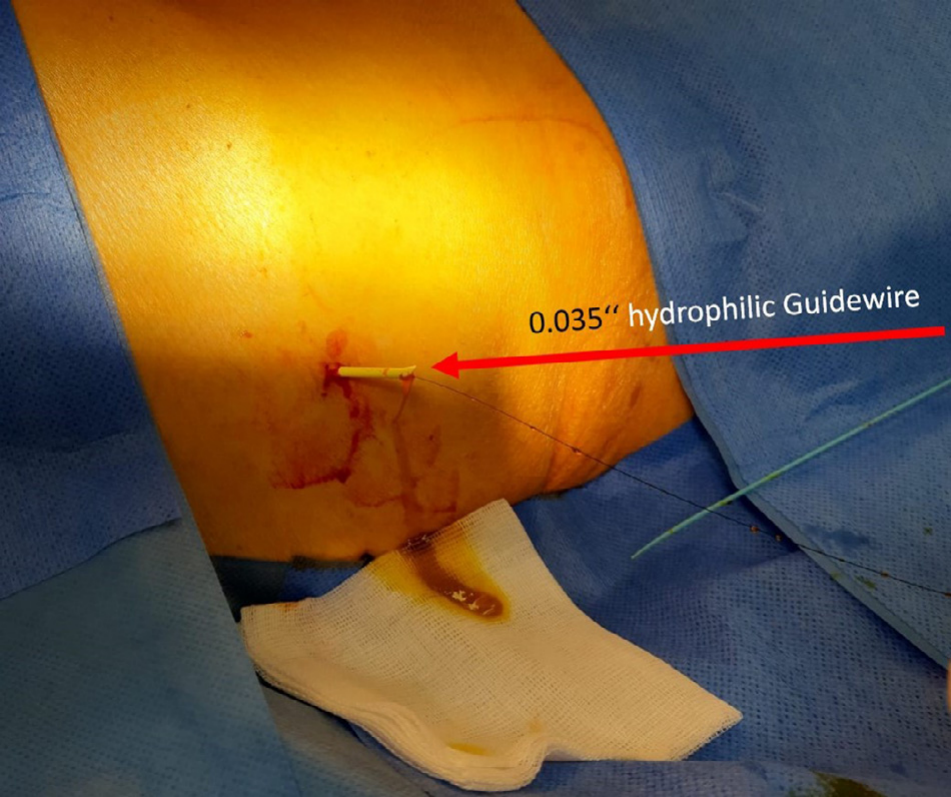
The threaded fPTBD was pulled outside of the body over the dilatator and an 0.035″ guidewire (Radifocus, Terumo Corp., Japan) was advanced over it.

**Fig. 8:**
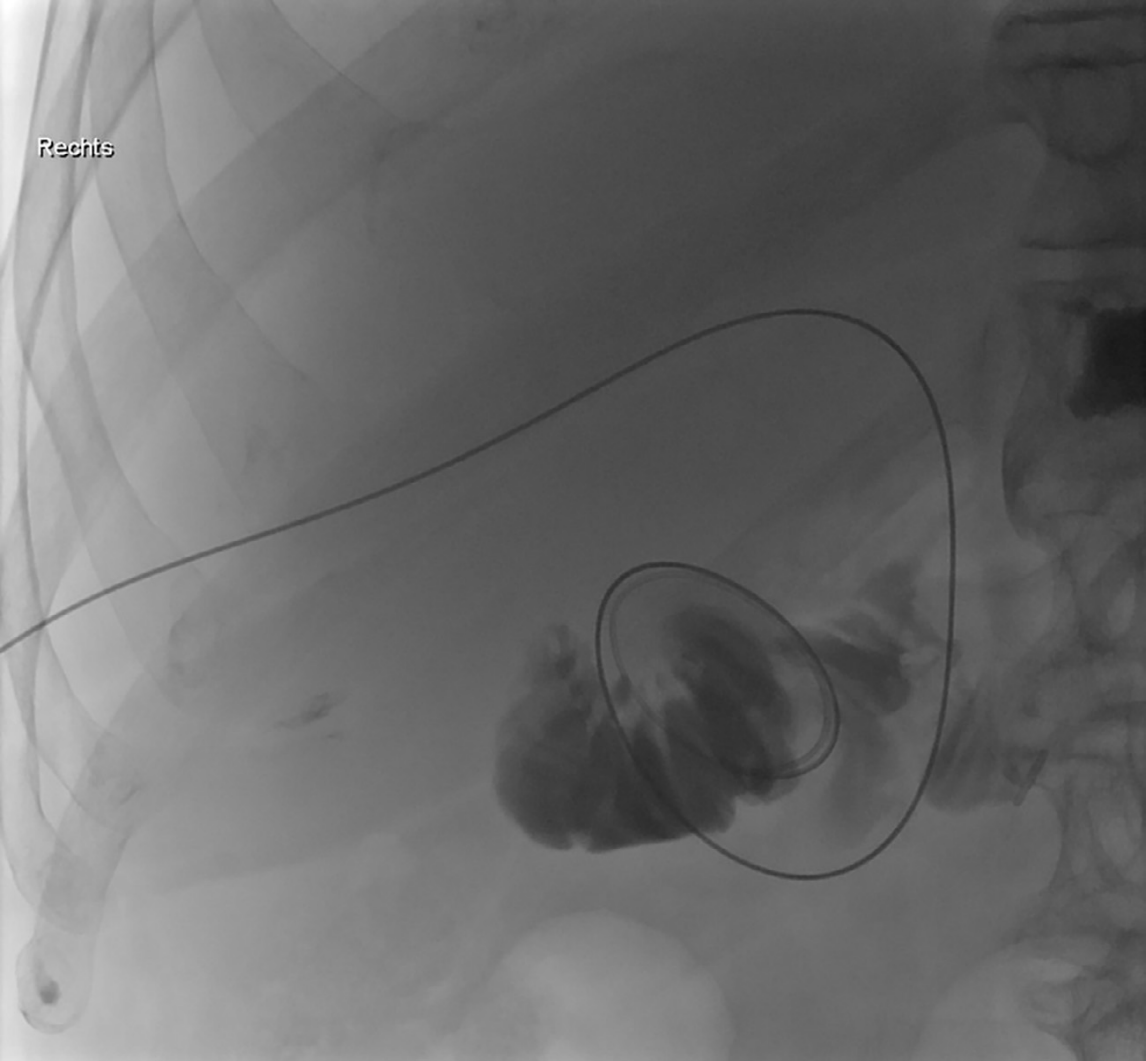
Fluoroscopic image showing the inserted 0.035″ guidewire (Radifocus, Terumo Corp., Japan) with its tip extending into the jejunum.

The next morning (day 6) the patient was presented to the IR team and then assessed in our angio suite. The extracorporal fractured part of PTBD was in the wound dressing, but the configuration thread of the remaining intracorporal fractured part of the PTBD (fPTBD) was still ending extracorporally and so we captured it. After application of local anesthetics around the old PTBD-tract, the fractured thread was passed through the dilator of a 4F sheath (Terumo Corp., Japan). The dilatator was then introduced over the tighted end of the thread to entrap the intraabdominal ending of the fPTBD. Then the dilatator was firmly pushed into the intraabdominal ending of the fPTBD (see Fig. 4, Fig. 5, Fig. 6). After dissection of the overlying skin the dilator and the threaded fPTBD were pulled outside of the body. The thread and the dilatator were removed and an 0.035″ guidewire (Radifocus, Terumo Corp., Japan) was advanced over it with its tip ending in the jejunum (see Figs. 7 and 8).

**Fig. 9:**
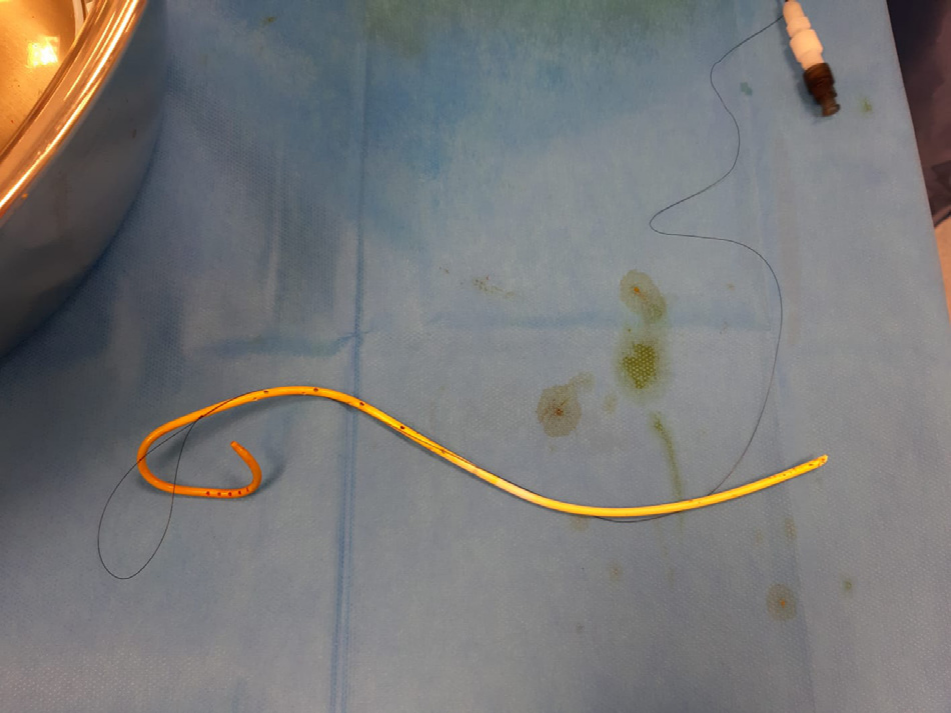
The fractured PTBD was then carefully removed over the inserted guidewire along with its fractured thread.

**Fig. 10:**
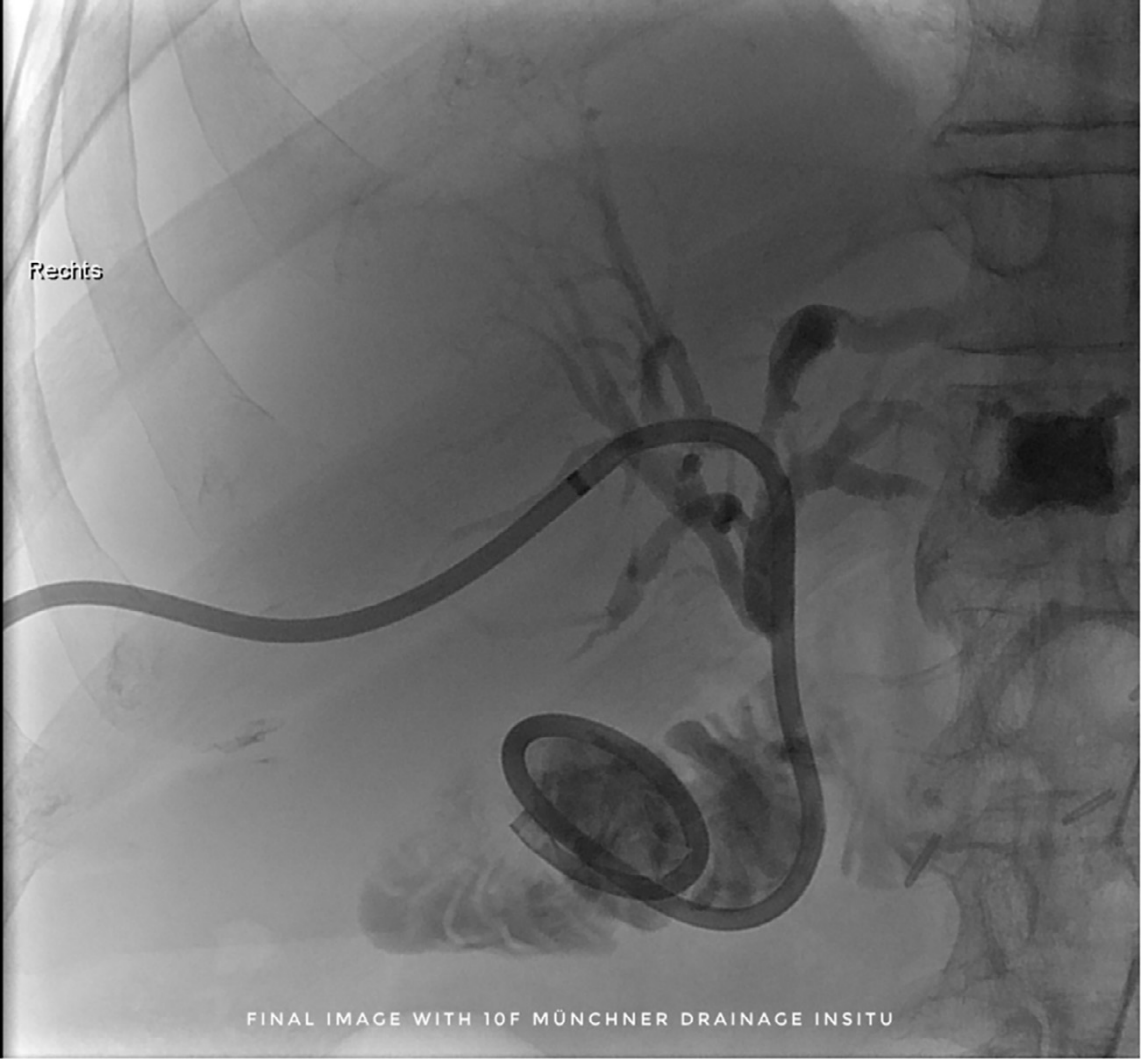
After switching to a 0.035″ heavy-duty wire (Amplatz Super Stiff™, Boston Scientific, USA), a new 10F PTBD was then placed.

The fPTBD was then removed and a multipurpose catheter (Glidecath, Terumo Corp., Japan) was advanced over the guidewire to secure the position (see Fig. 9). After changing to an 0.035′’ heavy-duty wire (Amplatz Super Stiff™, Boston Scientific, USA) a new 10F PTBD was then placed in position (see Fig. 10).

The bile secretions taken from the PTBD showed positive evidence of Pseudomonas aeruginosa and Escherichia coli. Therefore a therapy based on antibiogram by means of a combination drug with 4 g piperacillin and 0.5 g tazobactam (Actavis Group PTC, Norway) was started. Furthermore, a control multiphase contrast-enhanced (MDCT, Revolution HR, GE Healthcare, USA) of the abdomen was performed, which revealed a progressive intrahepatic biloma. The following morning (day 8) a CT-guided percutaneous drainage was placed for optimal biliary relief.

After a discussion with the patient's relatives to limit therapy due to progressive dementia, the gastroenterologists used PTBD for a rendezvous maneuver and were able to place the stent (noncovered, removable 10/40 WallFlex) endoscopically with good deployment and effective bile drainage. The patient was eventually discharged after an extended recovery period.

## Discussion

RPC is a chronic infective process of the biliary tree, presenting primarily with recurrent attacks of acute bacterial cholangitis first described in 1930 [9]. Definitive treatment includes antibiotic therapy, biliary decompression with biliary tree mapping through ERCP to characterize the extent of the disease following an multidisciplinary approach (interventional radiologists, gastroenterologists, and surgeons) in form of stricture dilatation, biliary drainage, stone removal, biliary bypass, liver resection and transplantation [10]. The role of IR in RPC is well defined which aims to relieve obstruction jaundice and drain the infected bile through PTBD placement, as well as to buy time for further therapy planning.

Out of all the PTBD placement related complications, obstruction was found to be the most common cause [11] and breakage of the PTBD the least common. The rate of breakage of PTBD tubes varies depending on the study, but it is generally reported to be between 1% and 5% [7]. Another study reports that PTBD breakage was encountered 16 times in a total of 9 patients, but does not provide further information on risk factors [12]. Breakage can occur when bile salts cause the catheter to become hard and brittle [13], henceforth we advocate in changing the catheter every 6 weeks. Other factors that can increase the risk of PTBD breakage, includes the type of tube used, the size of the tube, the location of the tube, the patient's underlying medical conditions and the patient's activity level. One study suggests that the risk of catheter fracture is related to the location of the catheter and the tightness of the suture used to secure it [13].

Signs and symptoms of PTBD breakage includes pain at the tube site, fever, chills, yellowing of the skin and eyes (jaundice), nausea and vomiting, diarrhea and decreased appetite. Complications of such breakage may include but not be limited to bile leakage leading to massive bile peritonitis [14], bleeding [15] and occlusion which further emphasizes on its immediate management.

Although the success rate of retrieving fractured PTBD catheters is reported to be between 80% and 90% [7], there is no standard treatment approach or proper technique which describes its removal and is mostly depended on individual interventionalists. Some interventionalists prefer to leave fragments alone as long as they are not causing any symptoms [13]. In order to analyze whether there is consensus in the literature on the optimal technique for the removal of a ruptured PTBD, we conducted a literature review between 1980 and 2023 and were able to find only limited information. Retrieval of such fractured PTBD was mostly done by IR technique such as balloon catheter, coaxial loop snare application [16] and 7 F biopsy forceps [7]. Surgical interventions should be considered if all other methods fail and if there are life-threatening complications.

**Fig. 11:**
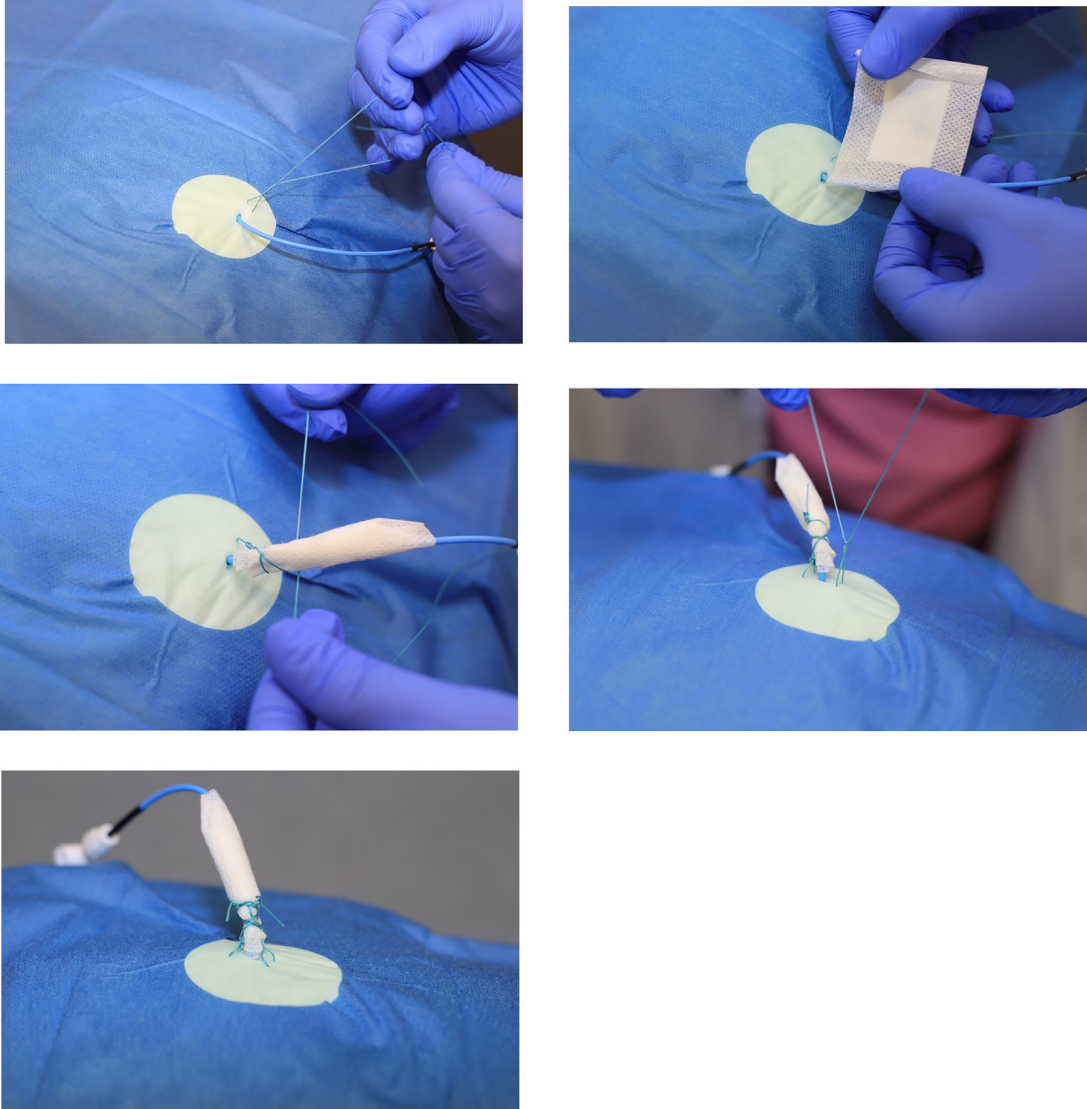
We recommend a double knot technique for fixing the PTBD subcutaneously, to prevent it from dislodging. A subcutaneous knot is created near the PTBD, leaving a 3 cm gap between the knot and the skin. Subsequently a Gauze is placed on the drain in place of the knot. A second subcutaneous knot is made using the same technique, and the suture is then tied and twisted into multiple loops to secure the gauze along with the PTBD. This method is repeated to get at least 7 to 8 knots.

In this case report, we describe a novel method of removing the fractured PTBD using a 4-French Dilator (Terumo Corp., Japan) which we used to entangle and capture the strings of the ruptured PTBD. In addition to this we also introduced a new technique to prevent this complication of subcutaneously fixing PTBD using the double knot-technique. By making a subcutaneous knot, leaving 3cm space between the knot and the skin to avoid local ischemia, a knot is then made over the lying PTBD to obtain a good grip. Gauze is then placed on the drain in place of the knot. A second subcutaneous knot is made using the same technique and the suture is now tied and twisted into several loops to secure the gauze. This procedure is repeated to achieve at least 7 to 8 knots (see Fig. 11).

## Conclusion

In conclusion the case highlights the role of the interventional radiology in handling a Fracture/breakage of PTBD with requires immediate management. Percutaneous approach should be preferred if ERCP fails, and surgical intervention should be considered if all other methods fail. Breakage and damage of abdominal drains can happen, especially in stuporose patients and a wide bandaging of the area with additional suturing of the extracorporal portion of the drain using the double knot-technique can help prevent such breakages.

## Patient consent

I state that written and informed consent was taken from the patient for publication of this case. The patient was informed that no personal details will be revealed in the publishing of this case.
